# Ophthalmic Formulation Containing Nilvadipine Nanoparticles Prevents Retinal Dysfunction in Rats Injected with Streptozotocin

**DOI:** 10.3390/ijms18122720

**Published:** 2017-12-15

**Authors:** Saori Deguchi, Hiroko Otake, Yosuke Nakazawa, Noriko Hiramatsu, Naoki Yamamoto, Noriaki Nagai

**Affiliations:** 1 Faculty of Pharmacy, Kindai University, 3-4-1 Kowakae, Higashi-Osaka, Osaka 577-8502, Japan; 1111610121m@kindai.ac.jp (S.D.); hotake@phar.kindai.ac.jp (H.O.); 2 Faculty of Pharmacy, Keio University, 1-5-30 Shibakoen, Minato-ku, Tokyo 105-8512, Japan; nakazawa-ys@pha.keio.ac.jp; 3 Laboratory of Molecularbiology and Histochemistry, Fujita Health University Institute of Joint Research, 1-98 Dengakugakubo, Kutsukake, Toyoake 470-1192, Aichi, Japan; norikoh@fujita-hu.ac.jp (N.H.); naokiy@fujita-hu.ac.jp (N.Y.)

**Keywords:** nanoparticle, nilvadipine, retina, electroretinogram, streptozotocin

## Abstract

Retinopathy leads to irreparable vision loss via capillary closure and areas of nonperfusion. However, the current instillation systems do not allow a sufficient amount of drug required to treat retinopathy to reach the posterior segment (retina); therefore, a new formulation targeting the posterior segment is expected as therapy for retinopathy. We prepared ophthalmic formulations containing nilvadipine nanoparticles (NIL_nano_), and demonstrated whether the instillation of NIL_nano_ can prevent retinal dysfunction in rats injected with excessive streptozotocin (STZ rats) in this study. NIL_nano_ (mean particle size, 77 nm) was prepared by wet bead mill treatment, with the inclusion of various additives (2-hydroxypropyl-β-cyclodextrin, benzalkonium chloride, d-mannitol, and methylcellulose). Retinal dysfunction was observable two weeks after rats received intraperitoneal injections of streptozotocin (100 mg/kg × 2, consecutive days, STZ rat). Changes in retinal function were evaluated by electroretinogram (ERG) and immunological methods. The retinal thickness, measured as the distance between the ganglion cell layer and the distal border of the outer nuclear layer, increased two weeks after the injection of streptozotocin, resulting in decreases in the levels of a-waves, b-waves, and oscillatory potential amplitudes in ERG of rats. The instillation of NIL_nano_ allowed the topical supplement of nilvadipine into the retina, and repeated instillation of NIL_nano_ (2 times/day) attenuated the retinal disorders led by the excessive streptozotocin. In conclusion, we found that retinal dysfunction in rats injected with streptozotocin can be prevented by the NIL_nano_ instillation. These results are useful in further studies aimed at the therapeutic treatment of retinopathy.

## 1. Introduction

Retinopathy comprises diseases in the posterior segment, such as glaucoma, diabetic macular edema, diabetic retinopathy, age-related macular degeneration, and proliferative vitreoretinopathy, and leads to irreparable vision loss. Drug delivery to the posterior segment has been broadly discussed in systemic and topical administration, although, the general ophthalmic formulations (eye drops, solution type) used clinically do not achieve sufficient drug levels into the posterior segment, since after instillation, drugs are removed by drainage through the nasolacrimal duct, tear turnover, and blinking. Moreover, the corneal epithelium acts as a barrier, and metabolism of drugs by anterior segment enzymes is also related to the bioavailability of topically administered agents. As a result, the amount of drug reaching the aqueous humor is only about 1% of the drug administered [1,2]. On the other hand, in the case of systemic administration, the blood–retinal barrier, inhibits the movement of systemically administered drugs to the retina. Therefore, it is necessary to administer high doses for delivery into the retina, which results in potentially serious toxicity and unwanted side effects. Within this context, the development of a drug delivery system to the posterior segment is greatly anticipated.

Drug administration by periocular injection (subtenon, peribulbar, retrobulbar, and subconjunctival injection) is now the method used to achieve high drug content into the posterior segment, and liposomes, micro- and nanoparticles, and transporter-mediated drug delivery systems have been evaluated as novel drug delivery systems for the posterior segment [3]. In particular, 50–200 nm of poly (dl-lactide-co-glycolide) (PLGA, biodegradable polymer) has been utilized as a drug delivery system for the posterior segment, and PLGA presents a possible solution to the limitations regarding ocular drug penetration [4,5,6,7,8,9,10]. We also previously designed an ophthalmic formulation containing drug nanoparticles (<100 nm), and showed that the installation of drug nanoparticles can deliver drugs into the posterior segment [11,12]. As stated above, these nanotechnologies show great potential for drug delivery.

Nilvadipine (NIL) is a dihydropyridine L-type voltage-dependent calcium channel (VDCC) blocker that increases vertebral blood flow more effectively than nifedipine or nicardipine, which are other calcium channel blockers of cerebral arteries [13]. It has been reported that NIL blocks Ca^2+^ influx via L-type VDCC in purified retinal ganglion cells, resulting in the inhibition of glutamate-induced apoptotic cell death [14], and the retinal ischemia–reperfusion injury [15]. In addition, NIL enhances blood flow and velocity in the retina, optic nerve head, and choroid in rabbits [16], and Hara et al. reported that the intravitreal endothelin-1-induced hypoperfusion of the optic nerve head was inhibited by the intravenous administration of NIL in rabbits [17,18]. These reports show that NIL may provide useful therapy for retinal diseases.

In this study, we designed an ophthalmic formulation containing NIL nanoparticles (NIL_nano_) produced by wet bead mill treatment, and investigated whether NIL_nano_ can normalize retinal dysfunction in model rats injected with excessive streptozotocin (STZ rats).

## 2. Results

### 2.1. Retinal Drug Delivery by Ophthalmic Formulations Containing NIL Nanoparticles

**Figure 1:**
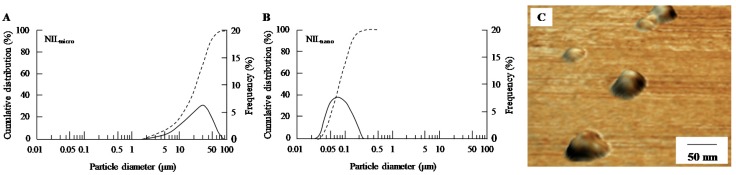
Size frequency distribution and images of 1% NIL microparticles (NIL_micro_) and nanoparticles (NIL_nano_). (**A**) and (**B**): cumulative size distribution and frequency of NIL_micro_ (**A**) and NIL_nano_ (**B**). Dashed line, cumulative size distribution; solid line, cumulative size frequency; (**C**) SPM image of NIL_nano_. The image was obtained with an SPM-9700. The NIL_nano_ (ophthalmic formulation containing NIL nanoparticles) was prepared by bead mill treatment, and the mean particle size was 77 nm.

**Figure 2:**
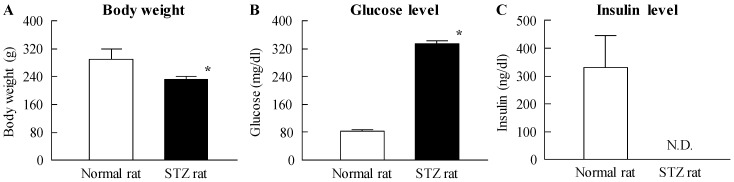
Body weight (**A**), plasma glucose (**B**), and plasma insulin (**C**) in normal and streptozotocin (STZ) rats. STZ rats were used two weeks after the injection of streptozotocin; blood was collected at 10:00 a.m. Open columns, normal rats; closed columns, STZ rats. *n* = 6. N.D., not detectable. * *p* < 0.05, vs. normal rat for each category. Hyperglycemia and hypoinsulinemia were observed in STZ rats two weeks after the injection of streptozotocin.

**Figure 3:**
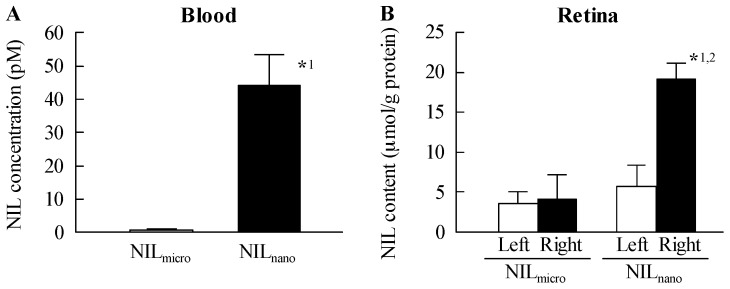
NIL concentrations in blood (**A**) and retina (**B**) of STZ rats instilled with NIL_micro_ and NIL_nano_. NIL_micro_ or NIL_nano_ was instilled into the right eye of STZ rats, and 50 min later, the rats were killed, and the blood and the right and left retinas were collected. NIL_micro_, NIL_micro_-instilled STZ rats; NIL_nano_, NIL_nano_-instilled STZ rats. *n* = 5. *^1^ *p* < 0.05, vs. NIL_micro_ for each category. *^2^ *p* < 0.05, vs. left retina (non-instilled eye) for each category. The NIL contents in retinas of rats instilled with NIL_nano_ were locally enhanced.

Figure 1 shows the size frequency distribution and images of ophthalmic formulations containing NIL microparticles (NIL_micro_) and NIL_nano_. The NIL microparticles were crushed with a bead mill, which reduced the mean particle size from 16 μm to 77 nm. Additionally, no aggregation or precipitation of NIL nanoparticles was observed for NIL_nano_ (mean particle size of NIL_nano_ two weeks after preparation was 82 nm). Figure 2 shows the changes in body weight and plasma glucose and insulin levels in rats injected with streptozotocin. The body weight and plasma insulin level in STZ rats were significantly lower than those of normal rats; the body weight of STZ rats was 80% that of normal rats. The plasma insulin level in STZ rats was below the detection sensitivity limit using a high sensitivity ELISA Insulin Kit (Morinaga Institute of Biological Science, Inc., Kanagawa, Japan; not detectable). In addition, the plasma glucose level was 4-fold higher in STZ rats than in normal rats. Figure 3 shows the transferability of NIL_nano_ to the blood and retina after instillation. Although the NIL concentration in the blood of rats instilled with NIL_nano_ was significant higher than when NIL_micro_ was instilled, the levels were still low. The NIL content in right retina (instilled eye) of STZ rats instilled with NIL_micro_ was similar to that in the left retina (non-instilled eye). On the other hand, the NIL content in the right retina (instilled eye) of STZ rats instilled with NIL_nano_ was approximately 3.4-fold higher than that in the left retina (non-instilled eye). Moreover, the NIL contents in the left retina (non-instilled eye) showed no significant difference between NIL_micro_- and NIL_nano_-instilled rats.

### 2.2. Effects of the Instillation of Nil_nano_ on Retinal Disorders in STZ Rats

**Figure 4:**
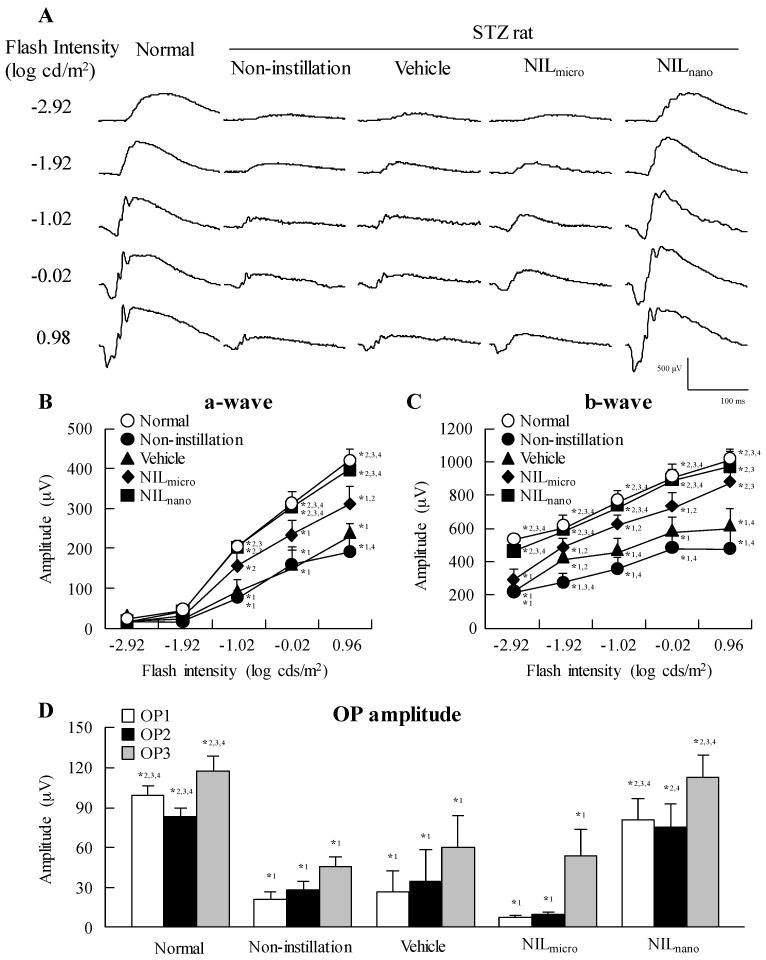
Typical traces of ERG (**A**); a-wave (**B**); b-wave (**C**); and OP amplitude (**D**) in STZ rats after the instillation of NIL_nano_. One percent NIL were instilled for two weeks (twice a day, 9:00 a.m. and 7:00 p.m.). Open symbols, normal rats; closed symbols, STZ rats. Normal, non-instilled normal rats; Non-instillation, non-instilled STZ rats; Vehicle, vehicle-instilled STZ rats; NIL_micro_, NIL_micro_-instilled STZ rats; NIL_nano_, NIL_nano_-instilled STZ rats. *n* = 5–7. *^1^ *p* < 0.05, vs. normal group for each category. *^2^ *p* < 0.05, vs. non-instillation group for each category. *^3^ *p* < 0.05, vs. vehicle group for each category. *^4^ *p* < 0.05, vs. NIL_micro_ group for each category. The retinal dysfunction of STZ rats was attenuated by the instillation of NIL_nano_.

**Figure 5:**
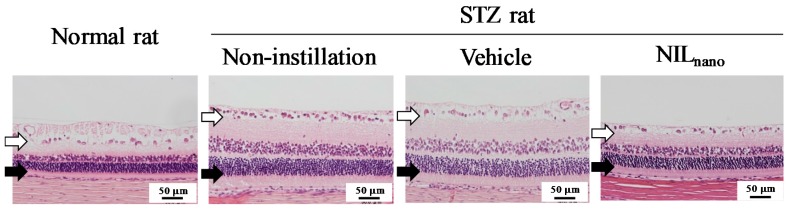
Preventive effect of NIL_nano_ on hypertrophic retina of STZ rats. Bars indicate 50 μm. One percent NIL was instilled for two weeks (twice a day, 9:00 a.m. and 7:00 p.m.). Open arrows, retinal ganglion cells. Closed arrows, outer granule layer. Normal rat, non-instilled normal rats; Non-instillation, non-instilled STZ rats; Vehicle, vehicle-instilled STZ rats; NIL_nano_, NIL_nano_-instilled STZ rats. The hypertrophy seen in STZ rat retinas was attenuated by the instillation of NIL_nano_.

Figure 4 shows the effect of NIL_nano_ instillation on ERG in STZ rats. Retinal dysfunction was expressed two weeks after the streptozotocin injection with the a-wave and b-wave levels and oscillatory potential (OP) amplitude in STZ rats significantly lower than normal rats. The instillation of vehicle and NIL_micro_ had no effect on the retinal dysfunction in STZ rats; however, the decreases in levels in a-wave, b-wave and OP amplitude were ameliorated by the instillation of NIL_nano_. The a-wave and b-wave levels and OP amplitude in NIL_nano_-instilled STZ rats were similar to those in normal rats. Figure 5 shows the changes in hypertrophic retina in STZ rats following the instillation of NIL_nano_, and Table 1 shows the retinal thickness measured as the distance between the ganglion cell layer and the distal border of the outer nuclear layer. Increases in the retinal thickness were observed in the retinas of STZ rats, and the retinal thickness in normal and STZ rats were 71.0 μm ± 3.57 μm and 130.6 μm ± 5.46 μm, respectively. On the other hand, the instillation of NIL_nano_ attenuated the increased the retinal thickness in the retinas of STZ rats.

## 3. Discussion

We prepared novel ophthalmic formulations containing NIL nanoparticles (NIL_nano_). The instillation of NIL_nano_ resulted in the presence of NIL in the retina, and attenuated retinal disorders caused by the injection of excessive streptozotocin.

We previously reported that drug nanoparticles, such as tranilast [19,20], indomethacin [21,22,23], cilostazol [24], dexamethasone [25], ketoprofen [26], ibuprofen [27], and disulfiram [28,29], can be prepared by bead mill treatment. Therefore, we attempted to design the NIL_nano_ based on our previous findings. Benzalkonium chloride (BAC), d-mannitol (mannitol), methylcellulose SM-4 (MC), and 2-hydroxypropyl-β-cyclodextrin (HPβCD) were used as additives in this study. BAC is the most common preservative in ophthalmic preparations, where it is often used clinically at concentrations <0.005%. Although BAC is necessary for the preparation of eye drops, BAC causes oxidative stress [30,31,32,33,34], and/or significantly alters precorneal mucins [35], resulting in corneal toxicity. On the other hand, we reported that the co-instillation of 0.5% mannitol attenuates the corneal toxicity of BAC [36]. Therefore, we used a concentration of BAC at 0.001%, to minimize its corneal toxicity, and added 0.5% mannitol to further prevent the toxicity of BAC. MC is a cellulose derivative, and our previous reports have shown that it enhances the milling efficiency in bead mill treatment; it is indispensable for the preparation of drug nanoparticles [19,20]. It is necessary to stabilize drug nanoparticles, and it has been reported that HPβCD prevents the cohesion of nanoparticulate solids by adsorption to the surface [23,37]. Based on these findings, 0.5% MC, 0.001% BAC, 0.5% mannitol, and 5% HPβCD were selected for the preparation of NIL_nano_. First, NIL microparticles were milled using the bead mill in the presence of the above additives, resulting in a reduction of the mean particle size from 16 μm to 77 nm (Figure 1). These NIL nanoparticles showed no observable precipitation seven days after bead mill treatment (mean particle size, 83 nm). Thus, we succeeded in preparing an ophthalmic formulation containing high-quality NIL nanoparticles.

Next, we investigated whether the drug was delivered into the retina by the instillation of NIL_nano_. The NIL content in the right retina (instilled eye) was increased (Figure 3B), and the NIL content in the left retina (non-instilled eye) of STZ rats instilled with NIL_nano_ was not different than the level of NIL_micro_ in the left retina of NIL_micro_-instilled STZ rats. These results show that NIL_nano_ can deliver the drug into the retina via topical pathways. In general, two topical pathways are known: (route I, non-corneal route) (1) conjunctiva, (2) sclera, (3) choroid/retinal pigment epithelium, and (route II, corneal route) (1) cornea, (2) aqueous humor, (3) intraocular tissues [38]. For drug solid nanoparticles, our previous study using cilostazol showed that the non-corneal and corneal pathways to both be involved in the delivery of nanoparticles to the posterior segment, such as retina [11]. Therefore, both pathways may be involved in the delivery of NIL_nano_. Further investigation is required.

We also demonstrated the preventive effect of NIL_nano_ instillation on retinal disorders in STZ rats. The loss of retinal neurons is observed early in the disease progression in both diabetic patients and diabetic animal models [39], and the ERG is found to be altered, even when there is no observed retinopathy in diabetic patients [40,41,42]. STZ rats have been used as a model of diabetic mellitus in various studies, and it is known that the development of retinal damage differs depending on the amount of streptozotocin injected. For example, at 8 weeks after the injection of streptozotocin (50 mg/kg), a-wave and b-wave responses are not significantly decreased, while there is a significant reduction in OP amplitude [43]. On the other hand, in the STZ rat injected with 60 mg/kg streptozotocin, the a-wave and b-wave amplitudes decrease two weeks after the injection. We also reported that apoptosis was not observed in the retina of rat model injected excessive streptozotocin, however, the a-wave and b-wave levels, in addition to the OP amplitude, are decreased in rats two weeks after the injection of excessive streptozotocin (100 mg/kg × 2 consecutive days, ip), and these changes in ERG are caused by the increase in the retinal thickness [44]. In addition, we showed that STZ rats injected with excessive streptozotocin can be used to evaluate the therapeutic effect of drugs on retinal disorders [12]. In this study, both hyperglycemia and hypoinsulinism was observed in the STZ rats, and the a-wave, b-wave, and OP amplitudes in the STZ rats were significant lower in comparison with normal rats. Moreover, an increase in the retinal thickness was observed. These results support a previous study on the condition of the retinas of STZ rats injected with excessive streptozotocin [12,44,45]. Furthermore, the instillation of NIL_nano_ prevented the changes in the ERG and retinal thickening in STZ rats (Figure 4 and Figure 5). These results are also similar to our previous reports; the ameliorants of the retinal circulation of cilostazol attenuated retinal disorders in STZ rats [12]. It has been reported that NIL increases the blood flow and velocity in the retina, optic nerve head, and choroid in rabbits [16], and suppresses the intravitreal endothelin-1-induced hypoperfusion of the optic nerve head in rabbits [17,18]. Taken together, NIL_nano_ may prevent retinal hypoxia by the ameliorants of retinal circulation, resulting in a decrease in ERG and retinal thickening. The preventive effect of retinal disorders in NIL_nano_ is strong in comparison with ophthalmic formulations containing cilostazol nanoparticles prepared in our previous study, since the a-wave (228 μV), b-wave (678 μV), OP1 (59.5 μV), 3 (77.6 μV) amplitudes and distance in the neural layer (81.8 μm) of STZ rats instilled with 1% cilostazol nanoparticles (instillation, twice a day for 2 weeks; flash intensity in ERG, −2.92 log cds/m^2^, *n* = 5) were lower than the results for NIL_nano_ (Table 1 and Figure 4) [12]. From these results, it is possible that NIL_nano_ may be an outstanding candidate for an ophthalmic formulation for the therapeutic treatment of retinopathy.

In this study, the NIL concentration in the blood of rats instilled with NIL_nano_ also increased, and the blood concentration was 44 pM after NIL_nano_ instillation (Figure 3A). It was known that the *C*_max_ is approximately 10 nM in humans upon administration with the commercial available 4 mg nilvadipine tablet, and the concentration 30 min and 8 h after administration is approximately 1.3 nM [46]. These results showed that the nilvadipine levels in blood after instillation was less than the NIL concentration that is therapeutically effective (positive effect), or that has adverse effects (side effects). On the other hand, the peripheral edema is known as the one side effect of nilvadipine, however, the instillation of NIL_nano_ showed local effects, and the peripheral edema in retina was not observed in the repetitive instillation (Figure 5). Further studies are needed to demonstrate the benefit in other aspects of retinopathy, and clarify the precise mechanism of the preventive effect of NIL_nano_ on retinal dysfunction. In addition, it is important to evaluate the periocular injection of NIL_nano._ Therefore, we are now investigating the effect of NIL_nano_ instillation and intravitreal injection on retinal disorders in the genetic model of diabetic retinopathy.

## 4. Materials and Methods

### 4.1. Reagents and Animals

NIL was purchased from Tokyo Chemical Industry Co., Ltd. (Saitama, Japan). BAC was obtained from Kanto Chemical Co., Inc. (Tokyo, Japan), and 0.4% Benoxil, 0.5% phenylephrine and 0.5% tropicamide were provided by Santen Pharmaceutical Co., Ltd. (Osaka, Japan). HPβCD and MC were kindly donated by Nihon Shokuhin Kako Co., Ltd. (Tokyo, Japan) and Shin-Etsu Chemical Co., Ltd. (Tokyo, Japan), respectively. SUPER FIX™ rapid fixative solution was purchased from Kurabo Industries, Ltd. (Osaka, Japan), and the ELISA Insulin Kit was obtained from Morinaga Institute of Biological Science, Inc. (Kanagawa, Japan). Mannitol and streptozotocin were purchased from Wako Pure Chemical Industries, Ltd. (Osaka, Japan). All other chemicals used were of the highest purity commercially available. Wistar rats aged 7 weeks were provided by Kiwa Laboratory Animals Co., Ltd. (Wakayama, Japan). STZ rats were prepared by injecting Wistar rats intraperitoneally with 100 mg/kg streptozotocin twice on two consecutive days, and then housing them for two weeks after the last injection before use in this study. All experiments were performed in accordance with the ARVO resolution on the use of animals in research, and were approved by the Kindai University Faculty of Pharmacy Committee Guidelines for the Care of Laboratory Animals (project identification code KAPS-25-003, 1 April 2013).

### 4.2. Preparation of Ophthalmic Formulation Containing NIL Nanoparticles

The ophthalmic formulation was prepared using aseptic techniques. Mixtures containing NIL powder (microparticles), MC, BAC, and mannitol were treated with a bead mill (3000 rpm, 30 s, 4 °C) in a tube with 2 mm zirconia beads, and the mixtures were added into saline containing HPβCD. The dispersions were then moved to new tube containing 0.1 mm zirconia beads, and crushed with the bead mill (5500 rpm, 30 s × 15 times, 4 °C). In this study, we used the milled dispersions as the NIL_nano_. The ophthalmic formulation containing NIL microparticles (NIL_micro_) was prepared by dispersing NIL microparticles into saline containing MC, BAC, mannitol, and HPβCD. The final compositions of the NIL_micro_ and NIL_nano_ dispersions were as follows: 1% NIL, 0.5% MC, 0.001% BAC, 0.1% mannitol, and 0.5% HPβCD, isotonization, pH 6.5. The particle size was determined by a SALD-7100 (Shimadzu Corp., Kyoto, Japan, refractive index 1.60-0.10i), and images were obtained with a SPM-9700 (Shimadzu Corp.).

### 4.3. High Performance Liquid Chromatography (HPLC) Method

The NIL concentration was determined by an HPLC method using a Shimadzu LC-20AT system (Shimadzu Corp.). The mobile phase was 50 mM phosphate buffer/methanol/acetonitrile (50/25/25, *v*/*v*, pH 7), and the flow rate of the mobile phase was 0.2 mL/min. The NIL was detected at 242 nm at 35 °C. Other conditions were as follows: column, Inertsil ODS (3 μm, GL Science, Tokyo, Japan); internal standard, 2.5 μg propyl *p*-hydroxybenzoate.

### 4.4. Measurement of Glucose and Insulin Levels in STZ Rats

Blood was drawn from the tail vein of STZ rats fasted for 15 h, and used to measure plasma glucose and insulin levels. The glucose and insulin levels were determined using an Accutrend GCT (Roche Diagnostics, Mannheim, Germany) and an ELISA Insulin Kit (dynamic range 0.1–6.4 ng/mL), respectively, according to the manufacturers’ instructions [47].

### 4.5. Measurement of NIL Concentration in the Blood and Retina

One percent NIL_nano_ (30 μL) was instilled into the right eye of STZ rats (instilled eye). Fifty min after instillation, the rats were killed by injecting a lethal dose of pentobarbital. The blood and retina were collected, and the blood and homogenized retina in methanol on ice were centrifuged at 9100× *g* for 15 min at 4 °C. The concentrations of NIL in the supernatants were determined by the HPLC method described above.

### 4.6. Measurement of ERG

One percent NIL was instilled into the right eye (instilled eye) of STZ rats for 2 weeks (twice a day, 9:00 a.m. and 7:00 p.m.), and the ERG readings were obtained by PuREC (Mayo, Inazawa, Aichi, Japan). Prior to measurement, the STZ rats were kept in a completely dark room for 24 h, and the pupils were dilated with 0.5% phenylephrine and 0.5% tropicamide. The golden-ring electrode, reference electrode, and neutral electrode (Mayo) were set to right cornea (instilled eye), tongue, and subcutaneously near the tail, respectively. Flash ERG was recorded in the dark room (all procedures were performed under dim red light), and a-wave, b-wave, and oscillatory potentials (OPs, OP1, OP2 and OP3) were analyzed. The OPs were isolated by the band pass filter, and OP amplitudes were measured using ERG with all frequencies (0.3–500 Hz).

### 4.7. Hematoxylin and Eosin (H.E.) Staining of the Retina

One percent NIL was instilled into the right eye (instilled eye) of STZ rats for two weeks (twice a day, 9:00 a.m. and 7:00 p.m.), after which the rats were killed by injecting a lethal dose of pentobarbital. The right eyes (instilled eyes) were removed, and fixed in SUPER FIX™ rapid fixative solution, and 3 μm paraffin sections were prepared and stained with hematoxylin and eosin (H.E.). The photographed area is determined according to our previous report (approximately the middle part of the optic nerve and the peripheral part of the retinal nerve) [12]. The extent of retinal thickening was determined by measuring the distance between the ganglion cell layer and the distal border of the outer nuclear layer. The distance was analyzed by Image J.

### 4.8. Statistical Analysis

All data are expressed as the mean ± standard error of the mean (S.E.), and unpaired Student’s *t*-test, Aspin-Welch’s *t*-test, or Dunnett’s multiple comparison was used for statistical analysis.

## 5. Conclusions

We prepared NIL_nano_ by bead mill treatment in the presence of various additives, and instilled the NIL_nano_ to deliver the drug into the retina via topical pathways. We found that the retinal dysfunction in rats injected with streptozotocin was attenuated by NIL_nano_ instillation. These findings provide significant information that can be used to design further studies aimed to find therapy for retinopathy.

## Figures and Tables

**Table 1:** Effect of NIL_nano_ on the retinal thickness of rats two weeks after the injection of excessive streptozotocin.

Group	Normal Rat	STZ Rat		
		Non-Instillation	Vehicle	NIL_nano_
Distance (µm)	71.0 ± 3.57 *^2,3^	130.6 ± 5.46 *^1^	132.5 ± 5.14 *^1^	77.7 ± 4.16 *^2,3^

One percent NIL was instilled for two weeks (twice a day, 9:00 a.m. and 7:00 p.m.) following the injection of streptozotocin. Normal rat, non-instilled normal rats; Non-instillation, non-instilled STZ rats; Vehicle, vehicle-instilled STZ rats; NIL_nano_, NIL_nano_-instilled STZ rats. *n* = 5. *^1^ *p* < 0.05, vs. normal rat. *^2^ *p* < 0.05, vs. non-instillation. *^3^ *p* < 0.05, vs. vehicle. The instillation of NIL_nano_ prevented the spread of cells in the inner plexiform layer, and the outer and inner nuclear layers (neural layer) of the retinas.

## Abbreviations

BAC	benzalkonium chloride
ERG	electroretinogram
H.E.	hematoxylin and eosin
HPβCD	2-hydroxypropyl-β-cyclodextrin
HPLC	high performance liquid chromatography
MC	methylcellulose
NIL	nilvadipine
NIL_nano_	ophthalmic formulations containing nilvadipine nanoparticles
OP	oscillatory potentials
PLGA	poly (dl-lactide-co-glycolide)
S.E.	standard error of the mean
STZ rat	streptozotocin-induced rat
VDCC	voltage-dependent calcium channel
